# Factors associated with delayed decannulation within 180 days after pediatric tracheostomy in the PICU: a retrospective cohort study

**DOI:** 10.3389/fped.2026.1880157

**Published:** 2026-08-05

**Authors:** Wenmiao Xu, Jingya Sun, Jian Ji, Jiansheng Zeng, Zheng Li, Quan Wang, Chaonan Fan, Suyun Qian

**Affiliations:** 1 Department of Pediatric Intensive Care Unit, Beijing Children's Hospital, Capital Medical University, National Center for Children's Health, Beijing, China; 2 Department of Pediatrics, Shunyi Maternal and Children's Hospital of Beijing Children's Hospital, Beijing, China

**Keywords:** decannulation, long-term outcomes, pediatric intensive care unit, risk factors, tracheostomy

## Abstract

**Introduction:**

Long-term decannulation outcomes after pediatric tracheostomy, particularly among children discharged from the pediatric intensive care unit (PICU), remain poorly described. This study aimed to describe the long-term decannulation outcomes and factors associated with delayed decannulation within 180 days after hospital discharge.

**Methods:**

We retrospectively reviewed children aged 29 days to 18 years who underwent first-time tracheostomy in the PICU of Beijing Children's Hospital between January 2016 and December 2020. Follow-up data were obtained from medical records and standardized telephone interviews through August 31, 2025. The cumulative decannulation rate was estimated using the Kaplan–Meier analysis. Factors associated with delayed decannulation were analyzed using Cox regression. The discriminative ability of the independently associated factors in combination was assessed using receiver operating characteristic (ROC) curve analysis.

**Results:**

Among 234 children who underwent tracheostomy, 121 survivors with complete follow-up data were included in the final analysis. The median age at tracheostomy was 60 months (IQR, 15–111 months), and 30 patients (24.8%) were aged ≤1 year. At least one post-tracheostomy complication occurred in 66 children (54.5%), with late complications occurring in 62 children (51.2%). During final follow-up, 106 patients (87.6%) achieved successful decannulation, with a median time to decannulation of 126 days (IQR, 68–332). Within 180 days after discharge, 62 children (51.2%) achieved successful decannulation. Multivariable Cox regression analysis showed that age ≤1 year, obstructive airway disease, and poor cough reflex were independently associated with delayed decannulation within 180 days. The combination of these factors showed good discriminatory ability for 180-day decannulation status (AUC, 0.854; 95% CI, 0.783–0.926).

**Conclusions:**

Prolonged tracheostomy dependence was common after PICU discharge, with approximately half of children remaining cannulated at 180 days. Infancy, obstructive airway disease, and poor cough reflex were independently associated with delayed decannulation. Ventilator dependence and late complications were also associated with delayed decannulation. These late complications included respiratory infections, tube-related events, swallowing or phonation disorders, granulation tissue formation, tracheocutaneous fistula, and tracheal stenosis or malacia. These findings highlight the importance of individualized airway assessment, effective secretion management, surveillance for late complications, and continued multidisciplinary follow-up after discharge.

## Introduction

Tracheostomy is commonly performed in the pediatric intensive care unit (PICU) to relieve upper airway obstruction and facilitate prolonged mechanical ventilation (PMV) (1). With improvements in critical care, survival of critically ill children has increased, leading to a growing population requiring long-term tracheostomy care (2–4). However, decannulation before or shortly after discharge remains difficult in many cases, mainly due to persistent airway abnormalities, impaired secretion clearance, and complex comorbidities.

Prolonged tracheostomy use is associated with a range of complications. Previous studies have reported that recurrent respiratory infections, granulation tissue formation, tracheomalacia or tracheal stenosis, and swallowing or phonation dysfunction during long-term tube placement (5, 6). These complications may delay decannulation, increase healthcare utilization, and adversely affect quality of life (7, 8). Identifying children at high risk for prolonged tracheostomy dependence is therefore relevant to post-tracheostomy management.

Most existing studies on pediatric tracheostomy have focused on perioperative outcomes, mortality, or early complications (9, 10). Data on long-term decannulation patterns after PICU discharge remain limited, and factors influencing delayed decannulation are not fully defined. Therefore, this study aimed to describe long-term decannulation outcomes in pediatric PICU survivors following tracheostomy and to explore factors associated with delayed decannulation within 180 days after discharge. We also examined the potential influence of rehabilitation support on decannulation outcomes.

## Materials and methods

### Study design and ethical statement

This was a single-center retrospective cohort study conducted in the PICU of Beijing Children's Hospital, Capital Medical University. The study was approved by the Ethics Committee of Beijing Children's Hospital [(2024)-E-044-R]. Written informed consent was waived because of the retrospective study design.

### Study population and follow-up

Children aged 29 days to 18 years who underwent first-time tracheostomy in the PICU of Beijing Children's Hospital between January 2016 and December 2020 were screened. Patients were followed through August 31, 2025, using hospital medical records and standardized telephone interviews.

Information on survival status, decannulation status, post-tracheostomy complications, respiratory support, rehabilitation, and other long-term outcomes was obtained from medical records and follow-up interviews. Patients who survived to the end of the follow-up period and had available information regarding decannulation status were included in the final analysis. Patients with incomplete medical records or unavailable information regarding decannulation status were excluded.

The primary follow-up outcome was successful decannulation within 180 days after hospital discharge. Patients who remained tracheostomy-dependent at 180 days were classified as having delayed decannulation for the landmark analysis. For time-to-event analyses, the observation period began on the date of hospital discharge, and failure to achieve decannulation was defined as the event. Patients who had not undergone decannulation by the end of follow-up were censored at the date of the last available follow-up.

### Data collection

Clinical data were independently extracted from the Hospital Information System (HIS) and follow-up records by two investigators. Discrepancies were resolved by discussion with a third investigator.

Collected variables included (1) demographic characteristics, such as sex, age at tracheostomy, and body weight, (2) underlying diseases and primary indications for tracheostomy, (3) tracheostomy-related characteristics, including the interval between endotracheal intubation and tracheostomy, (4) respiratory support and airway-clearance characteristics, including mechanical ventilation status, prolonged mechanical ventilation, ventilator dependence at discharge, cough effectiveness, and swallowing function, (5) post-tracheostomy complications, including early and late complications, (6) post-discharge care and rehabilitation, including discharge destination and the availability of facility-based rehabilitation or structured rehabilitation support, and (7) decannulation outcomes, including decannulation during the index hospitalization, decannulation after hospital discharge, time to decannulation, and decannulation status at 180 days.

### Outcomes

The primary outcome was successful decannulation within 180 days after hospital discharge. Time to decannulation was defined as the interval from hospital discharge to successful removal of the tracheostomy tube.

Secondary outcomes included overall decannulation during the follow-up period and the occurrence of post-tracheostomy complications.

### Definitions

Underlying diseases were categorized as obstructive airway disease, neuromuscular, or cardiopulmonary disease.

Obstructive airway disease was defined as a structural or functional disorder causing clinically relevant upper or central airway narrowing or obstruction, including congenital or acquired airway stenosis, laryngotracheal abnormalities, tracheomalacia or bronchomalacia, and other documented airway lesions that impaired airway patency.

Cough effectiveness was assessed according to the response to airway stimulation, such as suctioning. An effective cough associated with adequate secretion clearance was classified as a good cough reflex, whereas a weak or ineffective cough with inadequate airway clearance was classified as a poor cough reflex.

The timing of tracheostomy was recorded as the interval between endotracheal intubation and tracheostomy. Early tracheostomy was defined as tracheostomy performed within 14 days after endotracheal intubation, whereas late tracheostomy was defined as occurring after 14 days.

Post-tracheostomy complications were categorized according to their timing and clinical characteristics. Early complications occurring were defined as complications occurring within 7 days after tracheostomy and included pneumothorax, air-leak syndrome, and bleeding. Late complications occurring after 7 days included respiratory infections, granulation tissue formation, tracheal stenosis or malacia, tracheocutaneous fistula, and swallowing or phonation disorders. Accidental decannulation and tube obstruction were recorded separately as airway-related adverse events regardless of the time of occurrence.

Prolonged mechanical ventilation (PMV) was defined as the requirement for mechanical ventilation for at least 6 h per day for a continuous period of 21 days or longer. A period of interruption of less than 48 h was considered part of the same continuous period of mechanical ventilation.

Prolonged PICU stay was defined as a hospitalization for more than 30 days.

### Sample size consideration

Because this was a retrospective cohort study based on a predefined clinical cohort, the sample size was determined by the number of eligible patients available during the study period rather than by prospective recruitment based on a prespecified effect size. All children who met the eligibility criteria during the study period were screened.

The final cohort included 121 survivors with complete follow-up information. Given the exploratory nature of this study, the sample size was considered sufficient to estimate the cumulative probability of decannulation and to explore clinically relevant factors associated with delayed decannulation. The number of events available for regression analysis was also considered when selecting the number of covariates for multivariable modeling.

### Statistical analysis

Statistical analyses were performed using SPSS version 23.0 (IBM Corp., Armonk, NY, USA). Categorical variables were presented as number (%) and compared using the Chi-square test or Fisher's exact test. Continuous variables were expressed as mean ± standard deviation or median with interquartile range (IQR), as appropriate. Student's *t*-test or Mann–Whitney *U* test was used for group comparisons.

Kaplan–Meier analysis was used to estimate the cumulative probability of decannulation after hospital discharge. The time-to-event variable was defined as the interval from hospital discharge to successful decannulation or the end of follow-up, whichever came first.

Univariable and multivariable Cox proportional hazards regression analyses were performed to identify factors associated with delayed decannulation. Delayed decannulation was defined as failure to achieve successful decannulation within 180 days after hospital discharge. Hazard ratios (HRs) and 95% confidence intervals (CIs) were calculated. In the Cox regression model, an HR >1 indicated a higher hazard of delayed decannulation, whereas an HR <1 indicated a lower hazard.

Variables with *P* < 0.15 in univariable analyses, together with clinically relevant variables, were considered for inclusion in the multivariable Cox regression model. The proportional hazards assumption was assessed before final interpretation of the model. The results of the multivariable Cox regression analysis were presented as a forest plot showing adjusted HRs and corresponding 95% CIs.

Because the primary clinical outcome was failure to achieve decannulation within 180 days after hospital discharge, receiver operating characteristic (ROC) analysis was performed to evaluate the discriminatory performance of the combined clinical model for 180-day delayed decannulation. The area under the curve (AUC) and corresponding 95% CI were calculated.

All tests were two-sided, and *P* < 0.05 was considered statistically significant.

## Results

### Baseline characteristics

**Figure 1:**
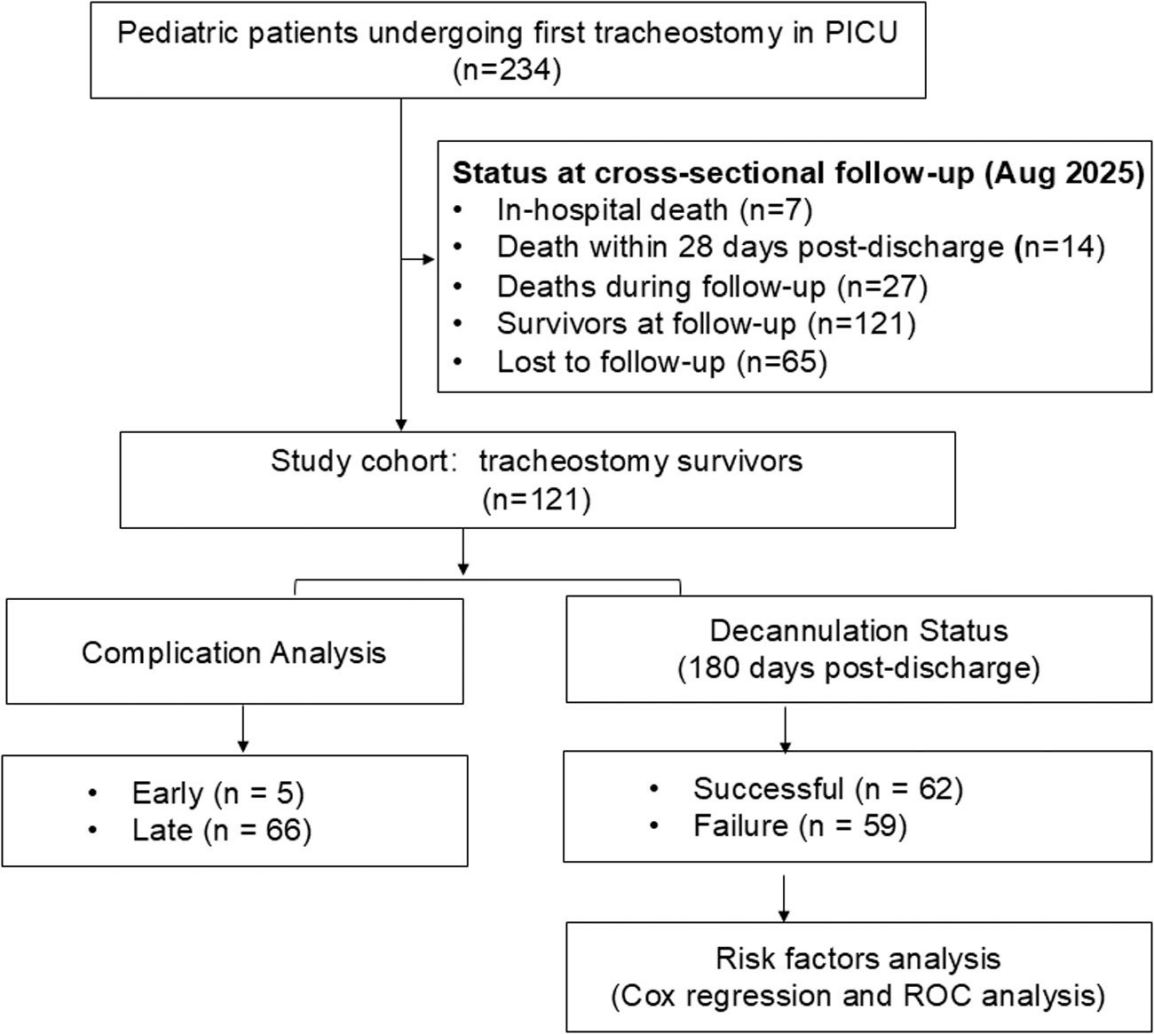
Study flowchart of the screening and follow-up process. This diagram illustrates the recruitment and attrition of the study population. Initially, 234 pediatric patients who underwent their first tracheostomy in the PICU were screened. Following a cross-sectional follow-up as of August 31, 2025, 48 deaths and 65 lost-to-follow-up cases were identified and excluded. The final analysis cohort consisted of 121 survivors with complete longitudinal data. For the primary outcome analysis, patients were categorized into the Decannulation Success group (*n* = 62) and the Decannulation Failure group (*n* = 59) based on their status at the 180-day post-discharge landmark.

A total of 234 pediatric patients who underwent first-time tracheostomy in the PICU were screened. At follow-up in August 2025, 48 patients had died, including 7 inpatient deaths, 14 deaths within 28 days after discharge, and 27 deaths during later follow-up. In addition, 65 patients were lost to follow-up. Finally, 121 survivors with complete outcome data were included in the analysis (Figure 1).

No significant differences in baseline characteristics were observed between the patients included in the final cohort and those lost to follow-up (Supplementary Table 1).

Among the included patients, 71 (58.7%) were male. The median age was 60 months (IQR: 15–111 months), and 30 patients (24.8%) were younger than 1 year. The median body weight was 18.0 kg (IQR: 9.2–29.0 kg).

Neuromuscular disorders were the most common underlying diseases (56.2%), followed by obstructive airway diseases (31.4%) and cardiopulmonary diseases (12.4%). Tracheostomy was performed for prolonged mechanical ventilation in 68.6% of patients and for anatomical airway obstruction in 31.4%.

Most patients underwent tracheostomy after 14 days of endotracheal intubation (81.0%). During hospitalization, 43.8% of primary caregivers participated in bedside care and received tracheostomy nursing training.

### Distribution of complications

Overall, 66 patients (54.5%) developed post-tracheostomy complications. Late complications were more common than early complications (51.2% vs. 4.1%). Early complications mainly included accidental decannulation or tube obstruction (4.1%) and air leak syndrome (2.5%). The most common late complications were respiratory infections (20.7%), accidental decannulation or tube obstruction (17.4%), and granulation tissue formation (14.9%) (Table 1).

**Table 1:** Types of complications and distribution of incidence rates.

Type of complications	Frequency (n)	Incidence rate (%)
Early complications		
Accidental decannulation/obstruction	4	4.1
Air leak syndrome	3	2.5
Simple pneumothorax	1	0.8
Late complications		
Infection	25	20.7
Accidental decannulation/obstruction	21	17.4
Swallowing and vocalization disorders	20	16.5
Granulation tissue formation	18	14.9
Tracheocutaneous fistula	10	8.3
Tracheal stenosis/malacia	9	7.4

The incidence rate (%) is calculated based on the total number of cases in the study population. Individual patients may have experienced more than one type of complication.

### Decannulation outcomes

**Figure 2:**
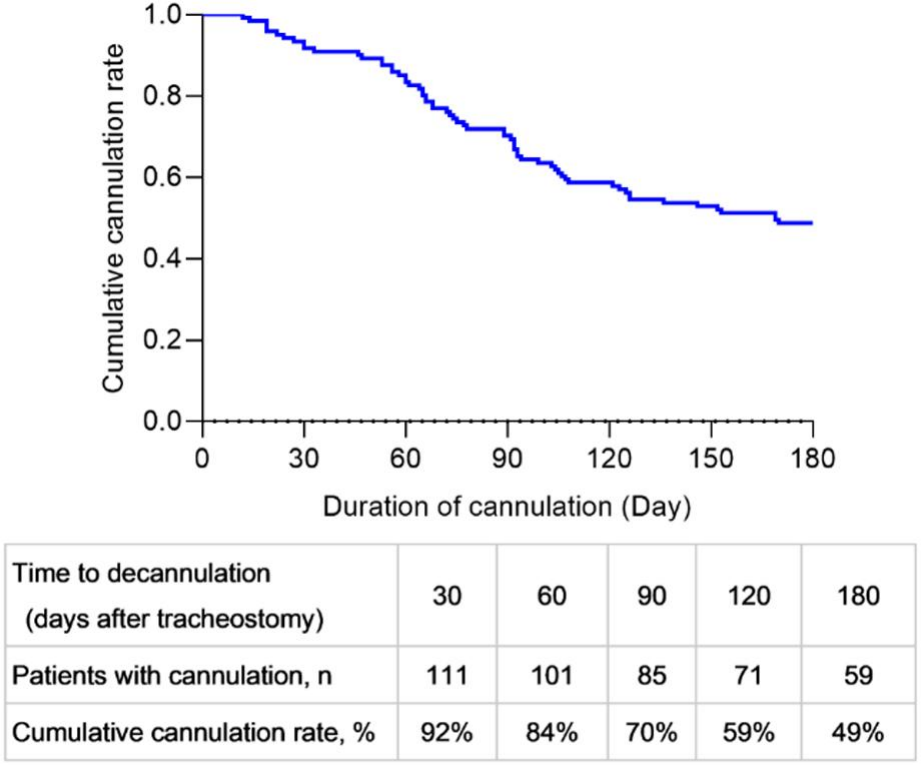
Cumulative cannulation rate following discharge from the PICU. The Kaplan–Meier survival curve demonstrates the dynamic proportion of patients remaining tracheostomy-dependent over a 180-day observation period. The *Y*-axis represents the cumulative cannulation rate, while the *X*-axis indicates the time (days) post-discharge. Tick marks denote censored data. The cumulative decannulation rate reached 51.2% by the 180-day landmark, with the cannulation rate dropping to 48.8% (rounded to 49% in the text). The table below the curve (if applicable) shows the number of patients at risk at key intervals (30, 60, 90, 120, and 180 days).

Only 10 patients (8.3%) were decannulated during PICU hospitalization. By the end of follow-up, 106 patients (87.6%) had achieved successful decannulation. The median time to decannulation was 126 days (IQR, 68–332 days). Within 180 days after discharge, 62 patients (51.2%) underwent successful decannulation (Figure 2).

### Factors associated with delayed decannulation

Univariable analysis showed that patients without decannulation within 180 days were younger than those successfully decannulated (median age: 32 vs. 82 months, *P* = 0.002). Obstructive airway disease (*P* < 0.001), ventilator dependence (*P* = 0.01), late complications (*P* = 0.001), and home care rather than institutional rehabilitation (*P* = 0.011) were also associated with delayed decannulation (Table 2).

**Table 2:** Analysis of factors influencing decannulation failure within 180 days after discharge in children with tracheostomy.

Variables	Non-decannulation (*n* = 59)	Decannulation (*n* = 62)	F/*χ*²	*P* value
Demographic characteristics				
Age (months), M (Q1, Q3)	32 (6, 92)	82 (47, 116)	9.801	0.002
Age group, *n* (%)			19.084	0.000
≤ 1 year	25	5		
＞1 year	34	57		
Gender (Male/Female)	38/21	33/29	1.559	0.268
Clinical features				
Obstructive airway disease, *n* (%)	31 (52.5)	7 (11.3)	23.881	0.000
Neuromuscular disease, *n* (%)	21 (35.6)	47 (79.7)	19.860	0.000
Tracheostomy timing (≤14 days), *n* (%)	34 (57.6)	27 (43.5)	2.397	0.122
Ventilator dependence, *n* (%)	14 (23.7)	4 (6.5)	7.126	0.010
Long-term complications, *n* (%)	40 (67.8)	22 (35.5)	12.634	0.001
Hospitalization & follow-up				
Long-term PICU stay, *n* (%)	18 (30.5)	23 (37.1)	0.586	0.565
Discharge destination (Home/Facility)	33/26	20/42	6.883	0.011
PMV at discharge, *n* (%)	26 (44.1)	38 (61.3)	3.559	0.043
Payment method (Self-pay/Insurance)	54/5	50/12	2.964	0.117
Duration of tracheostomy (days)	515 (283, 1,725)	75 (56, 105)	69.184	0.000
Duration of ventilator use (days)	194 (65, 317)	116 (30, 443)	2.436	0.121

Data are presented as *n* (%) for categorical variables and Median (Q1, Q3) for continuous variables unless otherwise specified. *F*/*χ*^2^ values represent the test statistics from independent samples *t*-test (or *F*-test/non-parametric equivalent) and Chi-square test, respectively. *P*-values < 0.05 are considered statistically significant.

PICU, Pediatric Intensive Care Unit; PMV, Prolonged Mechanical Ventilation.

**Figure 3:**
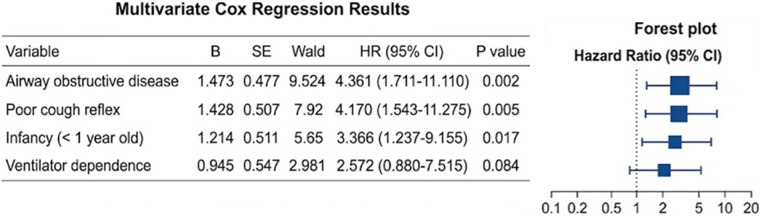
Forest plot of risk factors associated with decannulation failure within 180 days after discharge in children with tracheostomy. The left panel presents the results of multivariate Cox proportional hazards regression analysis, including hazard ratios (HRs), 95% confidence intervals (CIs), and *P* values. The right panel shows the corresponding forest plot, in which squares represent the point estimates of the HRs and horizontal lines represent the corresponding 95% CIs. The vertical dotted line indicates reference value (HR = 1). Associations were considered statistically significant when the 95% CI did not include 1 (*P* < 0.05). HR, hazard ratio; CI, confidence interval.

In univariable Cox regression analysis, obstructive airway disease, poor cough reflex, and age younger than 1 year were associated with delayed decannulation (all *P* < 0.05) (Table 3). In multivariable analysis, obstructive airway disease (HR = 4.361, *P* = 0.002), poor cough reflex (HR = 4.170, *P* = 0.005), and age younger than 1 year (HR = 3.366, *P* = 0.017) remained independently associated with delayed decannulation within 180 days after hospital discharge, whereas ventilator dependence (HR = 2.572, *P* = 0.084) was not independently associated with the outcome (Figure 3).

**Table 3:** Univariate Cox regression analysis of decannulation failure within 180 days after discharge in children with tracheostomy.

Variables	*B*	SE	Wald	HR (95% CI)	*P* value
Obstructive airway disease	1.639	0.403	16.565	5.152 (2.340–11.346)	0.000
Neuromuscular disease	0.435	.279	2.436	1.545 (0.895–2.667)	0.119
Dysphagia	0.396	0.297	1.782	1.486 (0.831–2.658)	0.182
Poor cough reflex	1.371	0.467	8.608	3.938 (1.576–9.837)	0.003
Infancy (<1 year old)	1.724	0.467	13.611	5.608 (2.244–14.015)	0.000
PICU stay >30 days	−0.141	0.263	0.288	0.868 (0.519–1.454)	0.591
Ventilator dependence	1.167	0.517	5.091	3.213 (1.166–8.858)	0.024
Institutional rehabilitation	−0.715	0.273	6.875	0.489 (0.287–0.835)	0.009
Long-term complications	0.936	0.266	12.353	2.551 (1.513–4.300)	0.000

B, Regression coefficient; SE, Standard error; HR, Hazard Ratio; 95% CI, 95% Confidence Interval; PICU, Pediatric Intensive Care Unit; PMV, Prolonged Mechanical Ventilation.

*P* values < 0.05 are generally considered statistically significant.

**Figure 4:**
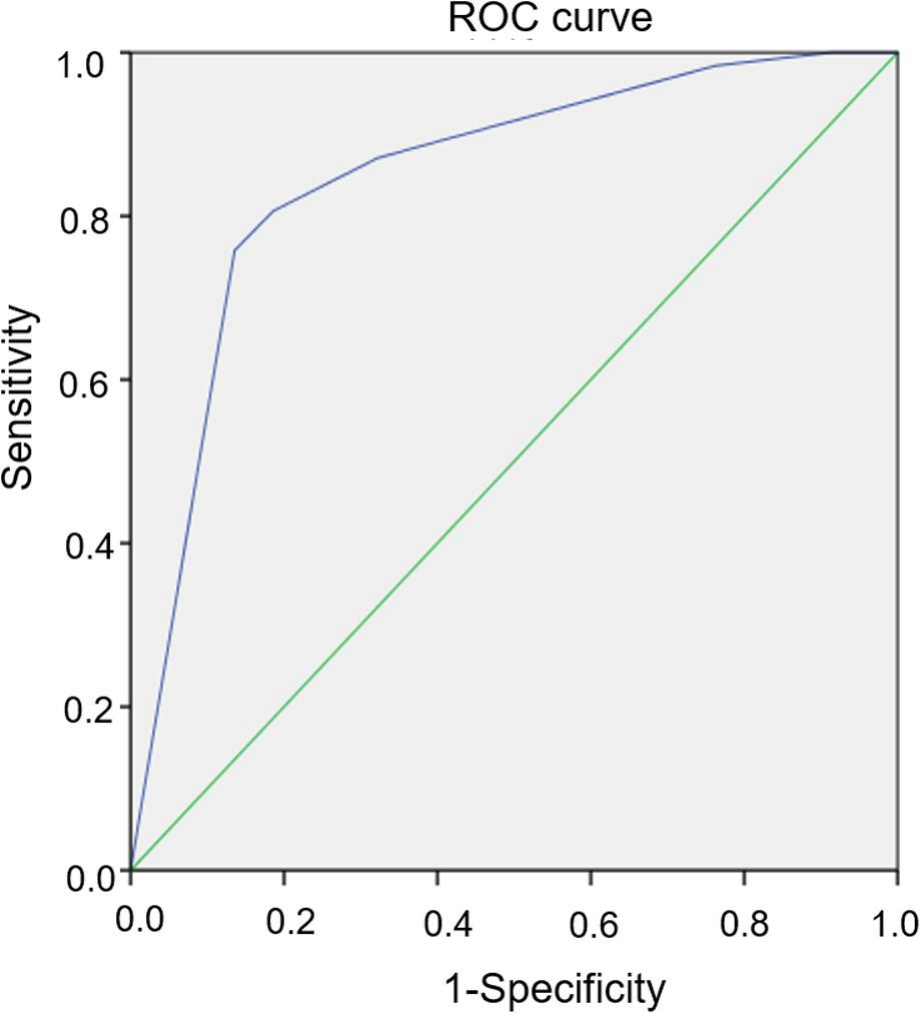
Receiver operating characteristic (ROC) curve of the combined predictive model for 180-day decannulation failure. The ROC curve evaluates the discriminatory performance of the multivariable model incorporating three independent risk factors: obstructive airway disease, age ≤1 year, and poor cough reflex. The combined model (blue line) achieved an Area Under the Curve (AUC) of 0.854 (95% CI: 0.783-0.926), indicating superior predictive accuracy compared to individual predictors. The diagonal green line represents the reference for a non-predictive model (AUC = 0.5). Sensitivity is plotted on the *Y*-axis and 1-Specificity on the *X*-axis.

The predictive ability (AUC) of the individual risk factors ranged from 0.629 to 0.706. The combined model showed better predictive performance, with an AUC of 0.854 (95% CI, 0.783–0.926) (Figure 4).

## Discussion

In this longitudinal follow-up study, nearly half of pediatric survivors who underwent tracheostomy in the PICU remained tracheostomy-dependent at 180 days after discharge. This finding suggests that recovery after pediatric tracheostomy is often prolonged and may be influenced by multiple factors, including airway pathology, respiratory function, and long-term care support.

Age ≤1 year was independently associated with failure of decannulation at 180 days. Infants have relatively immature airways with smaller airway diameters, lower respiratory reserve, and weaker airway support, which may increase the risk of airway collapse and respiratory insufficiency after decannulation (11). In addition, younger children often have less effective cough and secretion clearance, particularly during respiratory infections (12). Previous studies have similarly reported that younger age is associated with a higher risk of decannulation failure and the need for re-establishment of an artificial airway (13, 14). Our findings suggest that decannulation assessment in infants should extend beyond respiratory stability alone. Given their smaller airway caliber, limited respiratory reserve, and potentially less effective airway clearance, careful evaluation of airway patency, cough effectiveness, and secretion clearance may be particularly important before decannulation. These assessments may help identify infants who require further airway evaluation or targeted respiratory rehabilitation before attempting decannulation.

Obstructive airway disease was another important factor associated with prolonged tracheostomy dependence. Structural airway abnormalities, including congenital airway malformations, subglottic stenosis, and tracheomalacia, may persist even after the primary disease has improved, resulting in ongoing airway obstruction and impaired spontaneous ventilation (11). In contrast, some children with neuromuscular disorders may eventually achieve decannulation after neurologic recovery and rehabilitation (15, 16). This observation suggests that persistent structural airway lesions may have a greater influence on long-term decannulation outcomes than reversible neurologic impairment. Therefore, bronchoscopy and dynamic airway assessment may be particularly valuable before decannulation in high-risk patients (17–19).

Poor cough reflex was also significantly associated with unsuccessful decannulation. Effective cough is essential for airway protection and secretion clearance. Prolonged mechanical ventilation, recurrent respiratory infections, and neuromuscular impairment may all impair airway clearance in critically ill children (1, 3, 20). Ineffective secretion clearance increases the risk of mucus retention, recurrent infection, and atelectasis, which may delay decannulation (12). These findings underscore the importance of evaluating airway clearance function during the decannulation process. In selected patients, pulmonary rehabilitation, airway clearance techniques, and postural drainage may facilitate respiratory recovery.

Regarding complications, early postoperative complications were relatively infrequent and were generally manageable. In contrast, late complications were more common and mainly included respiratory infections, granulation tissue formation, and swallowing or phonation impairment. Prolonged tracheostomy tube placement may cause chronic airway irritation and inflammation, contributing to granulation tissue formation and airway remodeling (12, 21). Long-term airway exposure may also increase bacterial colonization and recurrent respiratory infections (22, 23). The incidences of granulation tissue, tracheal stenosis, and tracheocutaneous fistula in our cohort were comparable to those reported previously (24–26). These complications may delay decannulation and contribute to long-term airway morbidity. Notably, late complications were associated with decannulation failure in the univariable analysis, highlighting the importance of long-term airway surveillance and follow-up care (27).

Interestingly, children admitted to rehabilitation institutions showed higher decannulation success rates. This finding suggests that structured rehabilitation and specialized nursing care may promote recovery of respiratory and airway function (28). In contrast, limited home caregiving resources may increase the risk of complications and delay decannulation (29). These results further support the role of continuous multidisciplinary management in children with tracheostomies (30). Recent studies have shown that multidisciplinary airway teams may improve long-term outcomes and quality of care in this population (27, 30, 31).

Based on the identified risk factors, we developed a combined predictive model including age ≤1 year, obstructive airway disease, and poor cough reflex. The model showed good predictive performance, with an AUC of 0.854. Compared with individual clinical variables, the combined model may better reflect overall airway status and respiratory recovery, and may help identify children at high risk for prolonged tracheostomy dependence.

Several limitations should be acknowledged. First, this was a retrospective single-center study with a relatively small sample size, which may limit the generalizability of the findings. Second, only surviving patients were included in the analysis, potentially underestimating the overall burden of adverse outcomes after pediatric tracheostomy. Third, some functional parameters, such as respiratory muscle strength and dynamic airway measurements, were not routinely available. Prospective multicenter studies with standardized airway assessments are needed to validate these findings and further optimize long-term management strategies.

## Conclusions

Long-term decannulation outcomes among pediatric PICU survivors were influenced by multiple clinical factors, with approximately half of the children remaining tracheostomy-dependent 180 days after hospital discharge. Infancy, obstructive airway disease, and poor cough reflex were independently associated with delayed decannulation. Ventilator dependence and late post-tracheostomy complications were also associated with delayed decannulation in univariable analyses. The late complications observed included respiratory infections, tube-related events, swallowing or phonation disorders, granulation tissue formation, tracheocutaneous fistula, and tracheal stenosis or malacia. Careful assessment of airway patency and cough effectiveness, individualized secretion management, timely recognition and management of late complications, and continued multidisciplinary rehabilitation and nursing support may help guide decannulation planning and long-term follow-up in this population.

## Data Availability

The raw data supporting the conclusions of this article will be made available by the authors, without undue reservation.

## References

[1] Sachdev A Gupta N Singh BP Choudhari ND Sharma N Gupta S. Indication-based timing of tracheostomy and its effects on outcome in the pediatric intensive care unit. Pediatr Pulmonol. (2022) 57(7):1684–92. 10.1002/ppul.25952 35506424

[2] Abdelaal Ahmed Mahmoud M Alkhatip A Younis M Jamshidi N Hussein HA Farag E. Timing of tracheostomy in pediatric patients: a systematic review and meta-analysis. Crit Care Med. (2020) 48(2):233–40. 10.1097/CCM.0000000000004114 31939793

[3] Liu P Chen Y Yang Z Huang L Liu C Ren H. Impact of tracheotomy and its timing on outcomes in pediatric patients with prolonged mechanical ventilation: a multicenter study in China. Respiration. (2025) 104(12):894–905. 10.1159/000548148 40875770

[4] Pavone M Verrillo E Onofri A Caggiano S Chiarini Testa MB Cutrera R. Characteristics and outcomes in children on long-term mechanical ventilation: the experience of a pediatric tertiary center in Rome. Ital J Pediatr. (2020) 46(1):12. 10.1186/s13052-020-0778-8 32005269 PMC6995086

[5] Berry JG Graham RJ Roberson DW Rhein L Graham DA Zhou J. Patient characteristics associated with in-hospital mortality in children following tracheotomy. Arch Dis Child. (2010) 95(9):703–10. 10.1136/adc.2009.180836 20522454 PMC3118570

[6] Yousef A Soliman SI Solomon I Panuganti BA Francis DO Pang J. Impact of obesity on timing of tracheotomy: a multi-institutional retrospective study. Laryngoscope. (2024) 134(11):4674–81. 10.1002/lary.31586 38895915 PMC12011076

[7] Luna LLSS Duarte MDCMB Levy SSDS Lima LS. Clinical characteristics and gaps in palliative care among tracheostomized children: a retrospective observational study. J Pediatr (Rio J). (2026) 102(1):101480. 10.1016/j.jped.2025.101480 41320165 PMC12765322

[8] De Araujo OR Azevedo RT De Oliveira FRC Colleti Junior J. Tracheostomy practices in children on mechanical ventilation: a systematic review and meta-analysis. J Pediatr (Rio J). (2022) 98(2):126–35. 10.1016/j.jped.2021.07.004 34509427 PMC9432186

[9] Noy R Eytan D Cohen JT Ostrovsky D Shkedy Y Gordin A. Factors associated with early mortality in pediatric tracheostomy placement: a retrospective cohort study. Eur J Pediatr. (2024) 184(1):5. 10.1007/s00431-024-05830-x 39532724

[10] Morrison JM Steuart R Russell CJ. Pediatric tracheostomy-associated respiratory infections: an evolving paradigm. Curr Opin Pediatr. (2026) 38(3):308–16. 10.1097/MOP.0000000000001559 41944651 PMC13152059

[11] Fuller C Wineland AM Richter GT. Update on pediatric tracheostomy: indications, technique, education, and decannulation. Curr Otorhinolaryngol Rep. (2021) 9(2):188–99. 10.1007/s40136-021-00340-y 33875932 PMC8047564

[12] Powell J Powell S Mather MW Beck L Nelson A Palmowski P. Tracheostomy in children is associated with neutrophilic airway inflammation. Thorax. (2023) 78(10):1019–27. 10.1136/thorax-2022-219557 36808087 PMC10511973

[13] Kolb CM Halbert K Xiao W Strang AR Briddell JW. Comparing decannulation failures and successes in pediatric tracheostomy: an 18-year experience. Pediatr Pulmonol. (2021) 56(8):2761–8. 10.1002/ppul.25170 33200542

[14] Lloyd AM Behzadpour HK Rana MS Espinel AG. Time considerations and outcomes in pediatric tracheostomy decannulation. Int J Pediatr Otorhinolaryngol. (2024) 179:111934. 10.1016/j.ijporl.2024.111934 38537449

[15] Raynor T Bedwell J. Pediatric tracheostomy decannulation: what’s the evidence? Curr Opin Otolaryngol Head Neck Surg. (2023) 31(6):397–402. 10.1097/MOO.0000000000000929 37751378

[16] Lloyd AM Blumenthal DL Behzadpour HK Bauman NM Preciado D. Outcomes of infant laryngotracheal reconstruction in tracheostomy decannulation and avoidance. Int J Pediatr Otorhinolaryngol. (2025) 195:112452. 10.1016/j.ijporl.2025.112452 40609248

[17] El-Said H Price K Hussein A Ganta S Rao A Nigro J. Bronchial remodeling following airway stenting in pediatric patients with tracheobronchial and congenital heart disease. J Soc Cardiovasc Angiogr Interv. (2023) 2(5):101068. 10.1016/j.jscai.2023.101068 39132388 PMC11307877

[18] Dabbous H Chorney SR Johnson RF Kou YF. Surgical outcomes by early airway endoscopy findings after pediatric staged laryngotracheoplasty. Laryngoscope. (2024) 134(2):963–7. 10.1002/lary.30875 37458330

[19] Florentine M Civantos AM Luu K Jacobson L. The impact of a tracheostomy-safe bronchoscopy protocol on pediatric tracheostomy outcomes. Int J Pediatr Otorhinolaryngol. (2025) 199:112648. 10.1016/j.ijporl.2025.112648 41270329

[20] Chorney SR Beams DR Nadar A Afolabi F Gelfand A Brooks R. Bronchopulmonary dysplasia and ventilation-associated outcomes after pediatric tracheostomy. Pediatr Pulmonol. (2024) 59(12):3530–9. 10.1002/ppul.27247 39267435

[21] Griese M Felber J Reiter K Strong P Reid K Belohradsky BH. Airway inflammation in children with tracheostomy. Pediatr Pulmonol. (2004) 37(4):356–61. 10.1002/ppul.10432 15022133

[22] Lubianca Neto JF Castagno OC Schuster AK. Complications of tracheostomy in children: a systematic review. Braz J Otorhinolaryngol. (2022) 88(6):882–90. 10.1016/j.bjorl.2020.12.006 33472759 PMC9615521

[23] Pearce H Talks BJ Powell S Brodlie M Powell J. A systematic review of antimicrobial therapy in children with tracheostomies. Pediatr Pulmonol. (2024) 59(2):251–9. 10.1002/ppul.26766 38010838 PMC11497275

[24] Kawar L Clark E Kubba H. External peri-stomal skin granulations in paediatric tracheostomy: incidence, outcomes and a proposed treatment algorithm. Int J Pediatr Otorhinolaryngol. (2024) 176:111821. 10.1016/j.ijporl.2023.111821 38147731

[25] Young A Szymczak A Valika T Ghadersohi S Hazkani I. Prevalence, associations, and outcomes of suprastomal collapse after pediatric tracheostomy. Laryngoscope Investig Otolaryngol. (2025) 10(4):e70245. 10.1002/lio2.70245 40852584 PMC12369399

[26] Ha TN Jain S Schuman A Ongkasuwan J. Pediatric tracheotomy stomal maturation and tracheocutaneous fistulas. Laryngoscope. (2024) 134(6):2941–4. 10.1002/lary.31271 38265121

[27] Willis LD. Pediatric tracheostomy year in review. Respir Care. (2024) 69(8):1025–32. 10.4187/respcare.11932 38626953 PMC11298220

[28] Pizzuto MF Sutton AG Schroeder KS Bravo MA Li L Kihlstrom MJ. Characteristics and outcomes of patients discharged directly home from the pediatric intensive care unit. J Intensive Care Med. (2023) 38(8):737–42. 10.1177/08850666231162530 36895117 PMC10374988

[29] Aung WT Ong NY Yeo SQC Juhari NSB Kong G Lim NA. Impact of pediatric tracheostomy on family caregivers’ burden and quality of life: a systematic review and meta-analysis. Front Public Health. (2025) 12:1485544. 10.3389/fpubh.2024.1485544 39886387 PMC11780180

[30] Amin R Agarwal A Chiang J Collaco JM Cristea AI Propst EJ. Care of infants and children with tracheostomies: an official American thoracic society clinical practice guideline. Am J Respir Crit Care Med. (2025) 211(11):2001–20. 10.1164/rccm.202508-2055ST 41123183 PMC12618984

[31] Chorney SR Brown AF Brooks RL Bailey C Whitney C Sewell A. Pediatric tracheostomy outcomes after development of a multidisciplinary airway team: a quality improvement initiative. OTO Open. (2021) 5(3):2473974X211045615. 10.1177/2473974X211045615 34616995 PMC8488522

