# Supplementary material for: Factors associated with delayed decannulation within 180 days after pediatric tracheostomy in the PICU: a retrospective cohort study
Source: Front Pediatr. 2026 Aug 5;14:1880157. doi: 10.3389/fped.2026.1880157 (PMC13485925; doi:10.3389/fped.2026.1880157)
Supplement: Supplementary file 1 [file Table1.docx]

**Supplementary Table 1: Comparison of baseline characteristics between follow-up and lost-to-follow-up groups**

| Variables | Follow-up  (n=176) | Lost to follow-up  (n=65) | F/χ2 | P value |
| --- | --- | --- | --- | --- |
| Age (months) | 49 (12, 98) | 52 (38, 106) | 0.264 | 0.072 |
| Gender (Male: Female) | 100:58 | 39:26 | 0.212 | 0.377 |
| Timing of tracheostomy (days) | 15 (9, 20) | 15 (11, 22) | 0.003 | 0.554 |
| Tracheostomy timing group, n (%) |  |  | 0.036 | 0.483 |
| Early group | 89 (50.6) | 32 (49.2) |  |  |
| Late group | 87 (49.4) | 33 (50.8) |  |  |
| Primary etiology, n (%) |  |  | 5.032 | 0.081 |
| Airway obstruction | 55 (31.3) | 11 (16.9) |  |  |
| Cardiopulmonary disease | 22 (12.5) | 11 (16.9) |  |  |
| Neurological disease | 99 (56.3) | 43 (66.2) |  |  |
| PICU stay (days) | 25 (16, 43) | 27 (17, 43) | 1.561 | 0.261 |
| Duration of ventilation (h) | 469 (230, 798) | 528(314,903) | 2.656 | 0.065 |
| Total hospital stay (days) | 37(22,57) | 27(17,43) | 0.166 | 0.502 |
| Medical cost of ventilation (RMB) | 24135  (13972, 39267) | 23514  (14080, 33568) | 0.801 | 0.552 |

Data Presentation: Continuous variables are presented as median and interquartile range M (Q1, Q3). Categorical variables are expressed as frequency and percentage n (%).

Abbreviations: PICU: Pediatric Intensive Care Unit.

P < 0.05 was considered statistically significant.
